# Functional results following high tibial osteotomy: a review of the literature

**DOI:** 10.1007/s00590-017-2112-8

**Published:** 2018-01-04

**Authors:** Mark Webb, Varun Dewan, David Elson

**Affiliations:** 1 Northern Deanery, Waterfront 4, Goldcrest Way, Newburn Riverside, Newcastle upon Tyne, NE15 8NY UK; 2 Royal Wolverhampton NHS Trust, Wolverhampton Road, Heath Town, Wolverhampton, WV10 0QP UK; 3 Queen Elizabeth Hospital, Gateshead, NE9 6SX UK

**Keywords:** High tibial osteotomy, Osteoarthritis, Patient-reported outcome measures, Results, Review

## Abstract

**Introduction:**

High tibial osteotomy (HTO) is an operation used to treat patients with medial compartment knee osteoarthritis. The United Kingdom Knee Osteotomy Registry (UKKOR) has been set up to gather contemporaneous data on HTO throughout the patient journey. UKKOR uses a variety of patient-reported outcome measures (PROMs) to gauge the surgical outcome.

**Aim:**

The aim of this review is to analyse the published literature that has used PROMs to assess the outcomes following HTO.

**Methodology:**

Two searches of the literature were performed and compiled highlighting 95 articles of interest. After screening and manual additions, 23 manuscripts were reviewed and appraised using the appropriate Critical Appraisal Skills Programme Checklist (Kai et al. in PLoS Med 4(11):1766–1775, 2007).

**Results and discussion:**

There is a variety of published literature on HTO with a varied approach to the use of PROMs. Their use has increased recently, and studies have demonstrated that they are appropriate assessment tools for monitoring outcomes following HTO. In all of the studies that compared pre-operative to post-operative PROMs, there have been significant improvements. However, the data are varied due to differing study designs which in some instances have significant limitations.

**Conclusion:**

PROMs are effective ways to measure outcomes following HTO. They can also be useful in predicting outcome. The heterogeneity of the data and limitations of the study designs limit the transferability of the data. It is therefore important to analyse data from a multi-surgeon, multi-centre source that uses robust and constant pre- and post-operative PROMs.

## Introduction

High tibial osteotomy (HTO) is an operative technique for patients with medial compartment knee osteoarthritis. The aim of the procedure is to transfer the weight-bearing axis from the worn medial compartment of the knee laterally. This distributes more of the force through the unaffected lateral compartment. A surgical cut (osteotomy) is made through the proximal tibia, opening or closing a wedge. A device, such as a plate, is used to stabilise the osteotomy.

As the indications and techniques evolve, ongoing research is paramount to ensure patient selection is optimised [5, 10]. Patient-reported outcome measures (PROMs) help provide evidence for clinicians and managers to analyse the factors affecting the patients’ outcome [2, 25]. New registries should collect PROMs with the aim to improve patient care [1].

The aim of this literature review is to analyse and appraise the published literature focusing on using PROMs to assess the functional outcomes following HTO for the treatment of medial compartment knee osteoarthritis.

## Search strategy

Two unique structured searches were performed to identify the relevant published evidence. The primary search was performed during the development stage of the project in April 2016. The search structure and keywords are outlined in Table 1. A secondary search was performed in March 2017 at the time of finalising the study design and submission of the research proposal and ethics approval. The search structure and keywords are outlined in Table 2. The primary and secondary strategy used MEDLINE initially and was subsequently rerun in Embase and PubMed to identify the relevant publications.

**Table 1:** Primary search

**#**	Searches	Results
1	Exp knee/	12,116
2	Exp osteotomy/	28,190
3	Tibia$.tw.	61,347
4	2 and 3	3077
5	High.tw.	2,488,942
6	(High adj2 tibia$).tw.	1149
7	High tibial osteotomy.tw.	919
8	4 and 5	1037
9	6 or 7 or 8	1307
10	Osteotomy.tw.	19,658
11	6 not 10	160
12	9 not 11	1147
13	Function$.tw.	2,484,121
14	Result$.tw.	6,596,066
15	Outcome$.tw.	981,980
16	(Function$ adj2 result$).tw.	33,250
17	(Function$ adj2 outcome$).tw.	26,233
18	Prom$1.tw.	1863
19	Patient reported outcome measure$.tw.	1057
20	Patient related outcome measure$.tw.	38
21	or/16–20	60,399
22	12 and 21	61
23	From 22 keep 1–4, 6–10, 12–14, 16, 18–20, 22–24…	41
24	22 not 23	20
25	From 24 keep 2, 4	2
26	23 or 25	43
	Search rerun on Embase and Pubmed (1 unique results)	44

**Table 2:** Secondary search

#	Searches	Results
1	Osteotomy/and tibia.ti,ab.	1162
2	(High tibial and osteotom*).ti,ab.	1293
3	1 or 2	2297
4	Treatment outcome/	801,483
5	(Outcome* or PROM or PROMS).ti,ab.	1,288,804
6	Patient reported outcome*.ti,ab.	8512
7	(Functions adj2 result*).ti,ab.	39,998
8	(Functions adj2 outcome$).ti,ab.	35,714
9	or/4–8	1,846,851
10	3 and 9	875
11	Limit 10 to (humans and [“adult (19–44 years)” or “young adult and adult (19–24 and 19–44)” or “middle age (45–64 years)” or “middle aged (45 plus years)” or “all aged (65 and over)” or “aged (80 and over)”])	562
12	limit 11 to last 10 years	359
13	[Meta-Analysis as Topic/or meta analyS.tw. or metaanalyS.tw. or Meta-Analysis/or [systematic adj (review$1 or overview$1)].tw. or exp Review Literature as Topic/or (cochrane or embase or (psychlit or psyclit) or (psychinfo or psycinfo) or (cinahl or cinhal) or science citation index or bids or cancerlit).ab. or (reference list$ or bibliograph$ or hand-search$ or relevant journals or manual search$).ab. or [(selection criteria or data extraction).ab. and Review/)] not (Comment/or Letter/or Editorial/or [animal/not (animal/and human/)]]	229,321
14	12 and 13	4
15	Randomized Controlled Trials as Topic/or randomized controlled trial/or Random Allocation/or Double Blind Method/or Single Blind Method/or clinical trial/or clinical trial, phase i.pt. or clinical trial, phase ii.pt. or clinical trial, phase iii.pt. or clinical trial, phase iv.pt. or controlled clinical trial.pt. or randomized controlled trial.pt. ormulticenterstudy.pt. or clinical trial.pt. or exp Clinical Trials as topic/or (((clinical adj trial$) or ((singl$ or doubl$ or treb$ or tripl$) adj (blind$3 or mask$3))).tw. or PLACEBOS/or placebo$.tw. or randomly allocated.tw. or (allocated adj2 random$).tw.)) not (case report.tw. or letter/or historical article/)	1,390,246
16	12 and 15	56
	Search rerun on Embase and Pubmed (0 unique results)	56

**Fig. 1:**
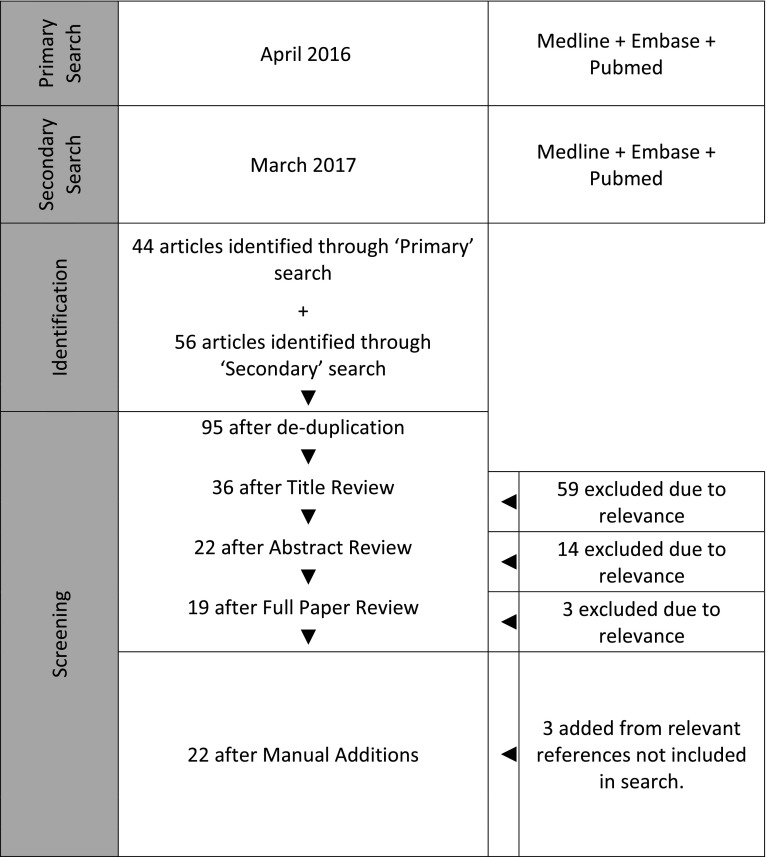
Literature search

The primary and secondary search results were combined and reviewed. Publications were eliminated if deemed irrelevant as summarised in Fig. 1. If there was uncertainty to the relevance of the article, it was included for full-text appraisal to avoid eliminating potentially significant evidence. During review of the publications identified in the literature searches, three were identified that had not been included in the search results. These were added for full-text review as illustrated in Fig. 1.

Full-text articles were reviewed and assessed using the appropriate Critical Appraisal Skills Programme Checklist [16]. Commentaries, operative techniques and editorials were excluded.

## Results and discussion

There is a plethora of publications commenting on a variety of topics around HTO. There is, however, little homogeneity in the use of reporting outcome measures. The majority of the literature is concerned with the degree of correction, complication rate, survivorship and rate of conversion to total knee arthroplasty (TKA). Those that report outcomes use a variety of measures.

### Patient-reported outcome measures (PROMs)

Patient-reported outcome measures are integral to determining the efficacy of current and novel treatments [25].

In 1986, Healy and Riley reported on 31 consecutive patients undergoing HTO. They used the Hospital of Special Surgery (HSS) Knee Score post-operatively and reported that 92% had an excellent result with 8% unchanged at 2 years. At 5 years, 88% continued to report post-operative improvement. Scores were not taken pre-operatively. Although this does not provide definitive evidence, as far as the authors are aware, this is the first mention of using patient-centred outcome measures in the literature [14].

A number of studies have used PROMs to compare different surgical techniques. Schallberger et al. used the Knee Injury and Osteoarthritis Outcome Score (KOOS) as well as Western Ontario and McMaster Universities Osteoarthritis Index (WOMAC) when retrospectively reviewing 71 patients. The aim was to present the long-term survival and outcome and determine if there was a difference between opening and closing wedge osteotomies. The authors reviewed a single surgeon’s cases from 1984 to 1992. Fifty-four patients were available. Kaplan–Meier survival analysis was performed [28].

Overall survival was 98% (CI 95–100%). At 5 years, survival was 92% (CI 86–99%) and at 10 years, 71% (CI 58–85%). KOOS score is 72 (IQR 49–82, range 9–100) and WOMAC 84 (IQR 66–96, range 9–100). There was no difference between opening and closing wedge osteotomies [28].

The authors report long-term follow-up data of a mean 16.5 years post-HTO. There was a 24% loss to follow-up. The authors note that these are now historical results and it is likely that, as surgical technique and hardware have developed, outcomes may have improved [28].

They were unable to comment on change from pre-operative function as this had not been completed [28]. This highlights the importance of establishing a prospective database.

Niemeyer et al. prospectively reviewed 69 patients over a 3-year period. The authors compared pre- and post-operative International Knee Documentation Committee (IKDC) data. A significant improvement was reported in the functional scores. There was improvement between 12 and 24 months and again between 24 and 36 months. This was thought to be due to implant removal which normally occurred after 24 months in their cohort [22].

A significant improvement in clinical outcome measured at 12 months was also noted by Schröter et al. in a prospective review of 35 procedures. They reported a statistically significant improvement in Lysholm, HSS and IKDC at 12 months compared with pre-operative scores. They also noted a significant increase in activity level measured using the Tegner score. These are listed in Table 3 [29]. Although only 35 cases were reviewed, the design of this therapeutic case series using multiple PROMs all demonstrating a significant improvement increases the validity of the findings. It provides level IV evidence that the scores are sensitive enough to detect a difference. The main weakness, other than sample size, is that this is one technique by one expert surgeon. Extrapolating these results to all surgeons should be done with caution.

**Table 3:**
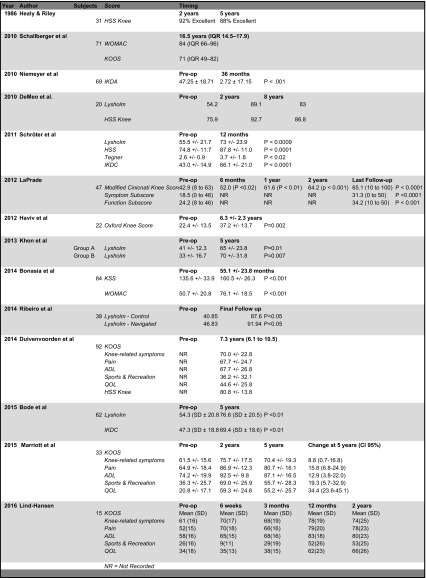
Summary of the literature

*NR* not recorded

DeMeo et al. reported on 20 consecutive patients over an 8-year period, noting that the HSS and Lysholm scores at 2 and 8 years had improved compared with the pre-operative scores. However, between year two and eight, there had been a decline in outcome score [7]. This is another case series supporting the use of PROMs in assessing post-operative improvement. With only 20 cases performed by an expert surgeon in a single unit, the results may demonstrate an exaggerated improvement.

Bode et al. reported on 62 patients who underwent HTO for medial osteoarthritis. IKDC and Lysholm were prospectively recorded pre-operatively. At 5 years, 11 patients were lost to follow-up (17.7%). Two of 51 underwent TKA resulting in a 96% survival in the patients that were followed up. Lysholm improved from 52.1 to 76.6. IKDC improved from 47.3 to 69.4 [3]. There was an 18% dropout rate which needs to be taken into account when interpreting the results of this level IV evidence.

Evidence for predictors of outcome has been published. Niinimäki et al. analysed the Finnish registry and found that overall survivorship of HTO, using TKA as the endpoint, was 89% at 5 years and 73% at 10 years. Males and those less than 50 years old had better outcomes [23].

This paper is not without limitations. The title suggests that there is an osteotomy database in Finland. In fact, the data were gathered by cross-referencing the Finnish National Arthroplasty Registry with hospital discharge data. They identified 3270 cases of which they had records, indicating that 1280 (39%) had undergone HTO. Seventy-five (2.3%) were excluded as they had undergone distal femoral osteotomy. The remaining 1915 did not have the type of osteotomy recorded and were assumed to have undergone HTO and included in the analysis [23]. If this assumption is incorrect, the data would be affected and invalidate the results.

Bonasia et al. reported significantly improved functional scores following the HTO (KSS and WOMAC) with > 56 years old and < 120 degrees of knee flexion pre-operatively negatively affecting the outcome. An excellent pre-operative KSS was a predictor of a good outcome [4].

Lind-Hansen et al. reported on a randomised control trial of three types of bone graft used during medial wedge HTO. Although the specifics are outside of the realms of this review, it did show an increase in KOOS across the domains at 1 year [19].

Ribeiro et al. reported the outcomes of 38 patients. Twenty were in the control group that underwent standard HTO and 18 in a group where the osteotomy was performed under navigation. Lysholm score improved significantly in both groups: control 40.85–87.60 and navigated group from 46.83 to 91.94 (*P* = 0.033). The greatest change was in the control group [26]. The study was performed from 2004 to 2012, and it is unclear from the paper at what point the follow-up Lysholm scores were undertaken. The authors also comment on the significantly higher score in the navigated group compared to the control group as an argument to use navigation. The greatest change in score was in the control group, however. This suggests that not using navigation may enable a greater absolute improvement. Although small numbers, this is evidence that HTO improves functional outcome which can be measured using PROMs.

A randomised control trial comparing opening and closing wedge HTO was reported by Duivenvoorden et al. Ninety-two patients were followed up for a mean of 7 years. The authors documented KOOS score at follow-up and showed that there was no difference between each of the groups in all domains. Pre-operative PROMs data were not gathered, and therefore, the authors were unable to comment on the degree of improvement [8]. This provides further support that PROMs are an effective way in measuring surgical and functional outcomes and can be used in randomised control trials to provide level I evidence.

Wu et al. performed a meta-analysis of 22 studies including seven randomised controlled trials and 15 non-randomised control trials. Four studies were removed due to sample duplication. Four studies used visual analogue score and showed no difference. Five studies used Lysholm and showed no difference. Range of movement was reportedly better in the opening wedge HTO group; however, a large sample from one paper may have influenced this [32]. Although this is a meta-analysis which in theory provides more robust analysis of the data, a major limitation in this review is that the studies included had a variety of PROMs used with differing methodology.

Marriott et al. performed a controlled laboratory study that assessed patients that had undergone HTO with ACL reconstruction. Gait analysis was performed as well as KOOS data gathered pre-operatively, at 2 and 5 years post-operatively. They reported statistically significant improvement in all domains of KOOS at 2 years which was maintained at 5 years [21]. The study involved 33 patients, and although the improvement is not solely due to HTO, it does demonstrate a positive effect in a well-conducted study. This is further evidence that KOOS is a sensitive tool to detect post-operative improvement.

LaPrade et al. collected modified Cincinnati Knee Scores (CKS) in patients less than 55 years old who underwent HTO for medial osteoarthritis and varus alignment. This was a single surgeon study from May 2000 to July 2007. The authors had tight inclusion and exclusion criteria which excluded patients undergoing additional procedures or treatments. Each patient was fitted with an offloading brace pre-operatively. If the patient did not get symptom relief, they were not offered a HTO [18].

Forty-seven patients were available for follow-up. The CKS improved from 42.9 to 65.1 (*P* < 0.0001). Function subscore improved from 24.2 to 34.2 (*P* < 0.001). Functional score improved significantly at 6 weeks, 1 and 2 years [18].

Limitations include the strict patient selection excluding patients that did not improve with the medial offloading knee brace. However, this may be a useful predictor and is something worth considering offering patients pre-operatively to maximise improved outcomes. The authors report a 20% loss to follow-up [18].

Haviv et al. reported on 18 patients with 22 HTOs. Procedures were performed for medial osteoarthritis and varus alignment in patients less than 65 years old. The procedures were performed by a single surgeon in a single institution. Oxford Knee Score (OKS) improved from 22.4 to 37.2 (*P* = 0.002) at a mean follow-up of 6.3 years. Age, BMI and gender did not affect the post-operative outcome [13]. This is a small case series using one surgical technique by a single expert surgeon. It has the same limitations as the other studies of a similar design.

Howells et al. reviewed 164 consecutive patients that underwent lateral closing wedge HTO between 2000 and 2002. One hundred patients met the inclusion criteria and were followed up at 5 and 10 years post-operatively. Data were collected prospectively; however, the study reviewed the data retrospectively. WOMAC and KSS were used to assess outcome [15].

Ninety-five patients were available for follow-up. At 5 years, there was an 87% survival rate with the remainder undergoing TKA. At 10 years, this dropped to 79%. It was noted that those requiring revision to total knee arthroplasty had a significantly lower WOMAC score (47 vs. 65 *P* < 0.001), were older (54 years old vs. 49, *P* = 0.006) and had a higher BMI (30.2 vs. 27.9, *P* = 0.005). It was concluded that a patient less than 55 years old, with a BMI less than 30 and a pre-operative WOMAC score of > 45, were positive predictors. The authors recommend the use of pre-operative functional scores to use in the decision-making process [15].

Although this study is based on patients undergoing lateral closing wedge HTO, the surgical principle is the same, to realign the weight-bearing axis. Therefore, these data support the use of PROMs in surgical planning. It did not comment on change in WOMAC scores at the time of follow-up and therefore cannot be used as evidence of improved function.

Kohn et al. [17] analysed their database to determine if age was a predictor to HTO outcome. They found 13 matched pairs of patients who had undergone medial opening wedge osteotomy. Group A had a median age of 57, while Group B had a median age of 42. VAS, Tegner and Lysholm outcome measures were used. There was no difference comparing pre- and post-operative Tegner scores, suggesting that activity levels did not change post-operatively. Lysholm (Group A: 41–65, *P* = 0.01, Group B: 33–70, *P* = 0.007) improved in both groups, but there was no significant difference between the groups. VAS improved in both groups (Group A: 77–36, *P* = 0.007 Group B: 73–41, *P* = 0.02). There was no difference between the groups [17].

Although this study has small numbers, the design and patient matching improve its validity in demonstrating improvement in both pain and function regardless of age.

### Return to activity

Return to sporting activities is an important consideration for many patients with early knee osteoarthritis. HTO provides an option for this group of patients as Bonnin et al. discuss. This retrospective review found that of the 139 patients reviewed 87 (63%) had normal knee function, while 86 (62%) noted that their knee limited their activities. Seventy-eight (56%) patients had matched their expectations, and of these almost all were satisfied with the outcome. Fifty-one per cent of patients who had not reached their expected level of activity were dissatisfied. Sixty-six per cent of motivated patients were able to return to strenuous activities; however, many patients continued to have symptoms [6]. Two hundred and sixty-seven patients were originally identified, but due to a number of exclusion criteria and loss to follow-up only 139 were questioned. Patients that did not respond to the questionnaire were excluded from analysis [15]. This discrepancy could significantly affect the results. However, the study involved patients operated on in four centres which provide data that are more relevant to day-to-day practice. The paper goes on to discuss the types of activities these patients are involved in and statistical analysis on the rate of activity. This analysis is interesting and demonstrates a methodology that future studies can build on. However, as scores and assessments were not completed pre-operatively there is significant risk of recall bias. As those who did not respond to the questionnaire were excluded from the study, the final group of patients by self-selection will tend to be motivated either by their positive or negative experience. This is borne out in the analysis which limits its ability to illustrate the true activity levels after HTO.

Ekhtiari et al. published a systematic review with the aim of examining timelines for return to sport and work following HTO and whether this was a comparable level to pre-operatively. The authors searched the literature and included papers that commented on return to work and/or sport. All athletic abilities were included. Patients were excluded if an additional procedure was performed alongside HTO or if an external fixator was used. The authors commented that patients with external fixators in situ would not be able to return to sport. This may be true, but it may not affect return to work [9].

Return to sport, in all studies, was deemed safe when the osteotomy had radiographically healed [9].

Two hundred and fifty patients in 11 studies were included. Eighty-seven per cent (87.2%) returned to sport with no comment on time from operation. In six studies, 89% returned to sport within 1 year with all patients returning to sport within 2 years. In 13 studies where level of sport was commented on, 78.6% of patients returned to sport at an equal or better level when compared with pre-operatively [15].

There were no consistent criteria for returning to work. Overall, 81.8% of patients returned to work post-operatively with 62.8% returning at an equal or better level. The authors commented that the studies examined a heterogeneous population and that return to work would be strictly guided by the type of work patients wanted to return to. Mean return to work was 3.5 months with more physical jobs taking longer. One paper examined military personnel who tended to return to work at a lower level. If this one paper was removed from analysis, 97.8% of patients returned to work at an equal or greater level. There were no reported complications due to early return to work or sport [9].

The paper included a wide variety of patient types, but there was a variety of activity scoring systems used which makes analysis challenging and can lead to error.

Gougoulias et al. reviewed the literature surrounding all lower limb osteotomies returning to sporting activities post-operatively. The authors found nine papers that found no difference in patients’ sporting activities following HTO when compared to their pre-operative level. They did note that no patients were playing at a competitive level [12]. This is a literature review that has a sound methodology.

Salzmann et al. published on patients’ sporting activity following HTO in 2009. This was level IV evidence. Eighty patients were sent a postal questionnaire including Lysholm score, Tegner Activity Scale, Activity Rating Scale and Visual Analogue Scale. Sixty-five patients returned the completed forms (83.1% compliance). The range of follow-up was 14–84 months with an age range of 19–65. The study is at risk of recall bias due to its retrospective design. The significant range in follow-up time could exacerbate this bias. As pre-operative scoring was not performed, the comparison was made to their perceived pre-operative function and symptoms.

The results showed that lifetime engagement in sports was 95.5% with 15.2% engaging in competitive sports. The year prior to surgery patients engaging in sports dropped to 87.9%. Post-operatively, there was no significant change in these figures. No patients competed competitively following HTO, and overall activity level was reduced. Thirty-five per cent reported a post-operative Tegner score of greater than five which indicates participation in heavy labour for work and competitive low-impact sports such as cycling [27, 30].

The design and limitations of this study mean that the results should be interpreted with caution.

Seventy-five per cent of patients following HTO did not require analgesia to participate in sport, while 22% required occasional analgesia. There was no link to the degree of osteoarthritis, gender, body mass index, anaesthetic score or alignment [27].

Lind et al. performed a case controlled clinical laboratory study of 11 patients. They showed that walking speed and gait analysis normalised to that of the control group following HTO. This is level III evidence resulting from a study with sound methodology. Although the numbers are small and the patients were operated on by a single surgeon, it demonstrates that HTO restores normal biomechanics which in theory should improve functional outcome [20].

Oberg reported on the functional outcome of patients following HTO and whether it matched their expectations. They examined 32 men and 25 women using the Functional Assessment System 6 and 12 months post-operatively. Statistically significant improvement was seen in 10 of 20 variables at 12 months. Forty per cent reached their expected activity level. The authors suggest a trial of rehabilitation programmes [24].

### Predictors of outcome

Obesity and smoking had been hypothesised to negatively impact on HTO outcomes. Floerkemeier et al. reported a multi-centre review of 533 patients that had undergone HTO. One operative technique was used. Seventy-two per cent of patients responded with a mean follow-up of 3.6 years. The number of reported complications was 32 which they stated was out of 533. This is an assumption that the 147 that did not respond to the questionnaires did not have any complications. This risks underestimating the complication rate [15].

Smoking did not appear to affect the functional outcome. The authors used OKS to assess this. A BMI of greater than 30 displayed a significantly lower OKS. Smoking plus obesity did not exacerbate this. Smoking did not affect complication rate or non-union rate. Absolute weight did not influence complication rate or OKS [11].

Bonasia et al. concluded that an excellent pre-operative KSS was a predictor of a good outcome [5]. Howells et al. concluded that a patient less than 55 years old, with a BMI less than 30 and a pre-operative WOMAC score of > 45, were positive predictors. The authors recommend the use of pre-operative functional scores to use in the decision-making process [15].

## Conclusion

The aforementioned published evidence has tended to be small sample sizes in single centres. Many are retrospective reviews, and the authors have used a variety of knee scores. The papers with larger numbers are not without limitations.

The published evidence suggests that there are improved outcomes following HTO in symptom management, function and returning to activity. However, the variety of study designs and PROMs used makes interpreting these results as a collective difficult and at risk of confounding variables.

There are good evidence and consensus amongst the published literature that PROMs have an important role in pre-operative counselling and planning.

The United Kingdom Knee Osteotomy Register (UKKOR) has been established to collect multi-centre, multi-surgeon data including details on the surgical procedure and standardised PROMs. The data are collected real time using approved software to simplify the data collection [31]. These measures will help reduce the limitations of prospective cohort studies while providing evidence with larger patient numbers and standardised outcome measures. Data from such a database will help to provide robust data to influence outcomes.
